# 

**Published:** 1887-01

**Authors:** 


### THE LOUISIANA STATE DENTAL ASSOCIATION.

The annual meeting of the Louisiana State Dental Association will be held in Tulane Hall, at New Orleans, La., on the 23d, 24th and 25th of February, 1887.

A cordial invitation is extended to the members of the profession throughout the States to attend.

No efforts will be spared to make our guests comfortable and the meeting interesting and profitable.

An opportunity to witness the Mardi Gras festivities will be afforded those who come, also, favorable railroad rates may be had at that time as the Mardi-Gras takes place the day before meeting. For further information, address

P. J. FRIEDRICHS, *Chr. Ex. Com.*
155 Carondelet Street, New Orleans, La.

### Editorial, Etc.

University of Maryland, Dental Department—Annual Commencement for 1887.—The classes in this Dental Department have become so large that separate commencements will hereafter be held; that for 1887, will take place on the 16th day of March next at the Academy of Music, Baltimore City.

The custom heretofore has been to hold the commencements of the Medical and Dental Departments of the University of Maryland at the same time and place. But the Graduates of the Dental Department will be so many this year that it becomes necessary to hold a separate commencement for each Department—namely, Medicine, Law and Dentistry.

The present graduating class is the largest ever known in this section of the country; and the same may be said of the matriculates for the present session of 1886-87. The many friends of the University of Maryland will be pleased at this announcement of the continued and unprecedented success of its Dental Department from its organization, which is an evidence of the reputation it sustains throughout the civilized world.

### *To Readers and Correspondents!*

Communications intended for publication, and exchange journals and papers should be addressed to the EDITOR OF THE AMERICAN JOURNAL OF DENTAL SCIENCE, 259 N. Eutaw St., Baltimore, Md. All letters relating to the business of the JOURNAL, or containing cash remittances, to be directed to Messrs. SNOWDEN & COWMAN. Articles for publication should be received by the editor at least three weeks previous to the first month on which the number in which it is designed for them to appear, is issued.

☞ The AMERICAN JOURNAL OF DENTAL SCIENCE will contain fifty pages of reading matter in each number, making a volume of about *Six Hundred* pages,

EXCLUSIVE OF ADVERTISEMENTS.

